# Effects of an Exercise-Assisting Mobile App (Osteoarthritis-Rehabilitation Assistant [O-RA]) on Rehabilitation Outcomes in Older Adults: Randomized Controlled Parallel Clinical Trial

**DOI:** 10.2196/80971

**Published:** 2026-04-15

**Authors:** Pajaree Sornmayura, Sintip Pattanakuhar, Napaschol Intapan, Krittipat Tragoolpua, Kaewkla Sroykabkaew, Rungkan Wangboon, Krittipong Wachirangkul, Sartita Wannarat, Jakkrit Klaphajone

**Affiliations:** 1 Department of Rehabilitation Medicine, Faculty of Medicine, Chiang Mai University, Chiang Mai, Thailand; 2 Department of Biomedical Engineering, Faulty of Engineering, University of Strathclyde, 106 Rottenrow East, Glasgow, G4 0NW, United Kingdom, 44 07464155436; 3 Department of Computer Engineering, Faculty of Engineering, Chulalongkorn University, Bangkok, Thailand; 4 The Prince Royal's College, Chiang Mai, Thailand

**Keywords:** knee, osteoarthritis, rehabilitation, motion analysis, mobile app, compliance

## Abstract

**Background:**

Mobile apps and biofeedback using motion analysis have both been used separately to increase compliance with exercise programs. We developed a mobile app, Osteoarthritis-Rehabilitation Assistant (O-RA), that uses motion analysis technology in the mobile app to assist older adults with performing a knee exercise program.

**Objective:**

This study aimed to evaluate the effects of the O-RA app on the compliance and correctness of the exercise program by older adults.

**Methods:**

We conducted an assessor-blind, parallel-design, randomized controlled trial with 40 older adults (aged 60 years or older) who had no symptoms and no diagnosis of knee osteoarthritis. Participants were divided into 2 groups: O-RA app (intervention) group and standard treatment (control) group. Both groups were taught 4 types of exercise programs by a physical therapist for 15 minutes and were instructed to do exercises at home every day for 1 week. The number of exercises, the percentage between observed and prescribed exercises, the correctness of exercises, and overall pain during the program were assessed in both groups.

**Results:**

The control group had significantly higher compliance with the exercise program than the intervention group (*t*_38_=3.5044, *P*=.001). There was no statistically significant difference in the correctness of the exercise program between the intervention and control groups. The difficulty of use and satisfaction were 47 and 59, respectively, out of the full score of 100. The main problems were the instability and the difficulty using the app.

**Conclusions:**

In older adults without knee osteoarthritis symptoms or diagnosis, the O-RA app was not a facilitator but a barrier to the lower extremity exercise program. An updated version, aiming to increase the stability and make it more user-friendly, should be developed; however, more comprehensive data, including qualitative user feedback and standardized usability metrics, will be needed to effectively guide its design.

## Introduction

Osteoarthritis (OA) is a disease caused by the gradual degeneration of the joints [1]. There are many risk factors for the disease, such as increasing age, genetics, joint injuries, and abnormal knee structures or deformities [2]. The pathophysiology of OA involves an increase in force acting on the articular cartilage, causing a change in the synovial membrane and subchondral bone [3]. Knee joints are most commonly involved in OA. Currently, the treatment approach for knee OA starts with nonpharmacological treatment followed by pharmacological treatment [4].

Exercise is the first-line conservative treatment for knee OA. A systematic review aiming to demonstrate the effects of exercise modalities found that several current guidelines for the treatment of OA of the knee, including the American College of Rheumatology; European Alliance of Associations for Rheumatology; Osteoarthritis Research Society International; and European Society for Clinical and Economic Aspects of Osteoporosis, Osteoarthritis and Musculoskeletal Diseases [4,5], recommend aerobic exercise and leg muscle strengthening exercises; exercises to strengthen and endurance of the lower extremities, especially the knee extensors; exercises to increase knee flexibility or range; and low-impact aerobic exercises as a first-line nonpharmacological treatment with strong supporting evidence [4]. Focusing on the exercises for strengthening the knee extensors commonly used in clinical practice, Zacharias et al [6] conducted a systematic review and meta-analysis of the literature on strengthening exercises and found that they could increase knee extensor and flexor strength. This systematic review supports the beneficial effects of strengthening exercises on the muscle strength of knee extensors and flexors in patients with knee OA, although clinical outcomes such as pain reduction and functional improvement require further evaluation in larger clinical trials.

Although strengthening exercises are beneficial for patients with knee OA, emphasis should be placed on adherence to the exercises. A previous study found that only 60.5% of patients performed the exercises correctly after being taught by a physician, and this rate decreased to 48.8% at the 4-week follow-up [7]. Increasing adherence to self-administered home exercises by older adults is important.

Mobile health (mHealth) has emerged as a promising approach in health care, with the potential to support diverse activities across diverse populations including health education, increasing access to health care services, improving prevention strategies, enhancing treatment delivery, and supporting public health programs [8]. In the context of musculoskeletal rehabilitation, mHealth apps have been developed primarily to support treatment strategies and improve adherence to prescribed exercise programs [9]. The Osteoarthritis-Rehabilitation Assistant (O-RA) app described in this study was designed with this specific purpose: to assist older adults with performing knee exercise programs correctly and consistently through motion analysis technology integrated into a mobile app.

Several studies have used mobile apps to improve exercise efficiency in patients with knee OA. For example, Alasfour and Almarwani [10] studied the effects of an Arabic mobile app on exercise adherence and knee pain in patients with knee OA compared with standard care. They found that the app could significantly increase exercise rate and reduce pain. In addition, Biebl et al [11] found that the joint angle of exercise, measured using a mobile app, was not inferior to the joint angle of exercise measured by a physical therapist. In addition, a study by Thiengwittayaporn et al [12] that studied the effects of a Thai mobile app on the accuracy of home exercise in patients with knee OA found that the group using the mobile app had higher exercise accuracy.

One of the advantages of using a mobile app is that it can include a motion analysis tool in its features. Motion analyses during exercise can be used as a biofeedback method to improve the clinical results of the exercise program [13]. However, only the study by Biebl et al [11] integrated motion analysis into the mobile app. The Prince Royal College School and Chiang Mai University jointly developed the O-RA mobile app using the technique of motion analysis of the body with automatic learning of artificial neural networks (deep learning) technology to analyze exercise posture to increase the accuracy. We hypothesized that the O-RA app can enhance the exercise program by improving the compliance with and correctness of the exercise program. However, a phase 1 study aiming to prove the safety and efficacy of the intervention in older adults without disease should be conducted before using the app with patients with knee OA.

The primary objective of this study was to evaluate the effect of the O-RA app on improving compliance with the exercise program by comparing the number of times the exercise program was completed and percentage of the observed and prescribed numbers of exercises completed between a group using the O-RA app and a group using standard teaching. The secondary objectives were to evaluate the (1) effect of the O-RA app on the correctness of physical exercise and (2) safety of the exercise program by assessing whether participants in the intervention group experienced increased pain, measured using a visual analog scale (VAS), compared with the standard teaching group.

## Methods

### Study Design

This study was a parallel-design, assessor-blinded, phase 1 randomized controlled trial based on the CONSORT-EHEALTH (Consolidated Standards of Reporting Trials of Electronic and Mobile Health Applications and Online Telehealth) guideline. All parts of the study were conducted at the Outpatient Clinic of the Department of Rehabilitation Medicine, Maharaj Nakorn Chiang Mai Hospital, from January 2024 through November 2024.

### Study Populations

Since this study was a phase 1 trial, the study population was older adult volunteers without knee pain. The inclusion criteria comprised of (1) age ≥60 years, (2) no previous formal instruction on physical exercises for knee OA, (3) the ability to participate in all study processes, (4) being able to read and write in the Thai language, (5) having a caregiver who can record the volunteers’ daily physical exercises for the entire study, and (6) voluntary participation in the study for both volunteers and caregivers. The exclusions criteria were (1) having limitations in visibility or understanding how to use the O-RA app system; (2) the presence of contraindications to exercise, including heart disease, diabetes, or uncontrolled high blood pressure; (3) having knee pain or knee deformity or limited knee extension of more than 10 degrees or inability to bend the knee more than 90 degrees; and (4) having balance problems, indicated by being unable to get up from a chair without using hands.

### Sample Size Calculation

The sample size was calculated based on the primary outcome, which was compliance with the exercise program (ie, the percentage of the observed number of exercises compared with the number of prescribed exercises) according to the research by Alasfour and Almarwani [10] and calculated using the formula for comparing two independent means [14]. With an effect size of 28.05, an SD of the overall group of 28.54, an α of .05, and a β of .10, the sample size of each group should be 17. After accounting for 20% compensation for participants lost to follow-up, the final sample size was 40 (20 participants per group).

### Study Protocol

We invited 40 older adult volunteers without knee pain by posting an announcement in Maharaj Nakorn Chiang Mai Hospital. Interested volunteers were contacted by the researchers, and an appointment was made to provide information and perform an assessment before participating in the research at the rehabilitation medicine outpatient examination room. In addition to a search within their medical history, they were asked about a history of chronic diseases and abnormal symptoms, including a physical examination to assess for abnormalities in the knee joints and balance, as well as to exclude those with contraindications for exercise.

After being invited to participate in the study and meeting the inclusion and exclusion criteria, participants were assessed for their ability to use the O-RA app correctly. If they were able to do so, they were asked to provide written informed consent. After that, the participants were randomized into either an intervention or control group using a computer-based simple randomization method. The randomization was conducted by a research assistant who was not involved in the exercise teaching and outcome assessment processes. The randomization sequence was stored in a sealed envelope in a locked drawer, and the assessor was blinded to it.

Volunteers were randomly divided into 2 groups: O-RA app group (intervention group) and standard teaching group (control group). Volunteers in the O-RA app group were taught how to use the O-RA app system on a dedicated smartphone on which the O-RA app had already been installed. They were then taught an exercise program for knee OA by a physical therapist for 15 minutes and were instructed to do the exercises using the O-RA app at home every day, 3 times a day for 1 week. The standard teaching group was taught an exercise program for knee OA by a physical therapist for 15 minutes and then instructed to do the exercises by themselves at home every day, 3 times a day for 1 week without using the O-RA app. Both groups were given an exercise logbook to record the number of exercises actually completed. The logbook was recorded by caregivers to ensure that the recording was not biased. After 1 week, the number of times and percentage of exercises in both groups were assessed using a specially developed assessment form and analyzed as completeness of the exercise program. Correctness of the exercise program was assessed by a rehabilitation physician in both groups. The score and percentage of correctness were then calculated. Pain during the exercise program was assessed using the pain VAS in both groups. Satisfaction with the use and ease of use were also assessed only in the O-RA app group. The researchers then conducted the statistical analyses to compare the completeness and correctness of the exercise program between the intervention and control groups.

### Exercise Program

**Figure 1.**
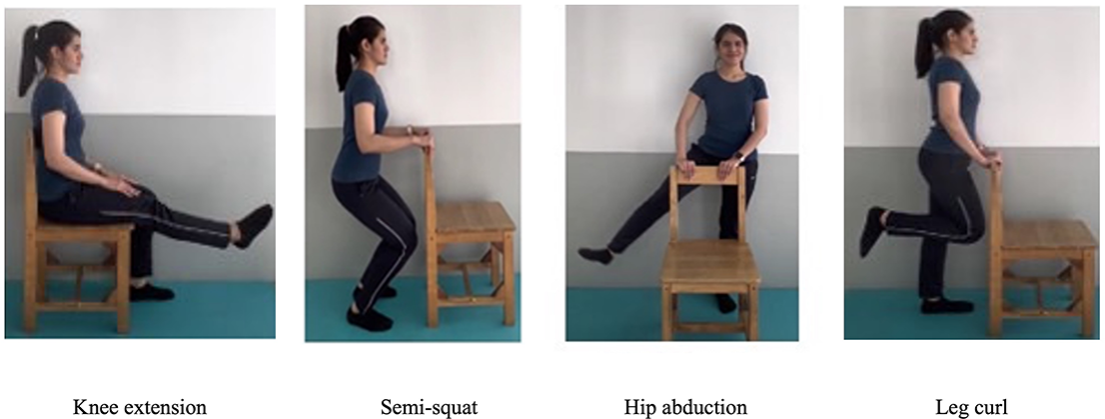
Exercise programs in the Osteoarthritis-Rehabilitation Assistant (O-RA) app. Written informed consent was obtained from the individual for the publication of any potentially identifiable images or data included in this figure.

The exercise program in this study consisted of 6 types of lower extremity exercises specifically designed by one of the investigators who was a specialist in sports rehabilitation (JK; Figure 1).

For the hip abduction exercise, the instructions were to stand up straight, hold the backrest of a chair or table edge, spread your legs about 30‐45 degrees to the side, perform one side at a time, and alternate the legs. The benefits are strengthening the hip abductors and improving stability while standing.

The leg curl instructions were to stand up straight, hold the backrest of a chair or table edge, and alternate bending one knee at a time. The benefits are strengthening the hamstrings and increasing stability while standing.

For the knee extension exercise, the instructions were to sit on the chair and alternate straightening one knee at a time. The benefits are strengthening the front thigh muscles and stretching the back thigh muscles.

The semi-squat instructions were to stand up straight, hold the backrest of a chair or table edge, slowly bend both knees until the knees are bent at about 45 degrees, and keep both feet on the ground. The benefits are strengthening the quadriceps and hip extensors and improving stability while standing; this exercise is more difficult than the knee extension.

### O-RA App System

**Figure 2.**
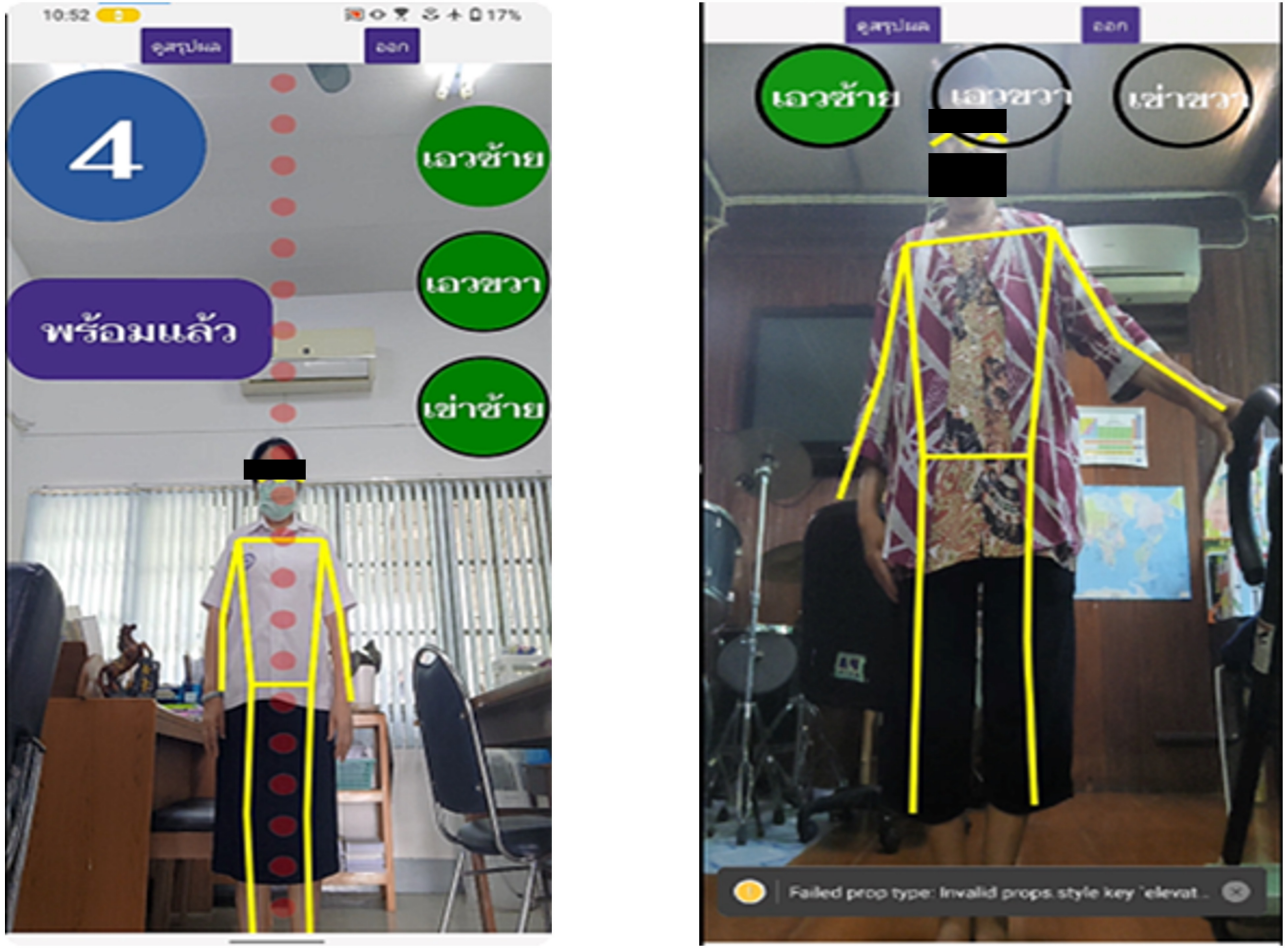
Example of how the app works while doing exercises.

The O-RA app is a mobile app available only on the Android operating system. The main function of the O-RA app system is to create motion analysis from a camera that is built into the mobile phone (Figure 2). To obtain this function, a pose estimation group of artificial intelligence was used to detect the joint position of the human body, process it into mathematical values, and apply an algorithm to calculate the degree of the joint movement. In this study, we used Movenet, a deep learning model for detecting points on the human body that can capture 17 joint points on the body. For the script programming language, JavaScript and cascading style sheets (CSS) were used. For the user experience (UX) and user interface (UI) design, the Figma collaborative interface design tool was used. The minimum requirements of the mobile phone for installing and running the O-RA app are as follows: central processing unit (CPU), Qualcomm Snapdragon 730 Octa Core; graphics processing unit (GPU) Adreno 618; 6 GB RAM; and 64 GB ROM. In this study, we used 2 mobile phones, both Samsung Galaxy S23 Ultra models (CPU, Qualcomm Snapdragon 8 Gen 2 Octa Core; GPU, Adreno 740; 8 GB RAM; 256/512 GB ROM). Firebase Storage from Google Cloud Platform was used as memory storage.

### Technical Development of the Physiotherapy Module

For the physiotherapy component, an algorithm was developed to assess whether the user’s exercise performance was correct. We used 3 adjacent keypoints from the MoveNet model output to calculate joint angles. First, the radian value was computed using the Math.atan2 function to determine the tangent of the angle (θ). This value was then multiplied by 180 and divided by π (3.1415), and the absolute value was obtained using Mathlabs, yielding the central angle between the 3 keypoints. After the model completed its prediction, it identified keypoints surrounding the center of the image, selecting the keypoint with the highest confidence score as the coordinate. Once MoveNet predicted the keypoint coordinates in the image, the developers fed the required coordinates into the mathematical formula to calculate the angle formed by 3 keypoints and verified whether the resulting angle met the physiotherapy criteria for correct exercise performance.

### Frontend Development (Mobile App)

The mobile app (Android) was developed using the React Native framework with JavaScript and CSS as the script programming languages. These languages enabled the integration of complex features into the mobile app, including smooth UX/UI design, user preference storage, and access to the mobile phone camera. React Native was selected because of its high performance in mobile environments and its structural similarity to the React framework, which facilitated the integration of the MoveNet model into the app. Additionally, the extensive and diverse package ecosystem of React Native provided the developers with multiple alternative solutions when encountering technical challenges during development.

For the app screen design, Figma was used as the collaborative interface design tool, enabling multiple users to work on the design simultaneously in real time and to export button graphics and other visual assets, which significantly streamlined the design process in React Native. For the in-app typography, the Fahkwang-Bold font was selected because its clear letterforms and distinct character heads facilitate readability for older adult users compared with standard fonts.

### Backend and Database

Firestore from Google Cloud Platform was used as the database because of its fast data storage, retrieval, and transmission capabilities. Firestore is cost-effective for small-scale applications, with no charges incurred when data storage and transfer volumes remain low while being easily scalable as the user base grows. Firestore also provides high security levels, with access rights that can be restricted to individual users or administrators, and data encryption before storage in the cloud database. Firebase Storage from Google Cloud Platform was used to store user profile images, selected for its seamless integration with Firestore and comparable security features. Firebase Authentication was used for the login and registration system because of its multilayered data encryption and user management capabilities.

### Speech Synthesis

For speech synthesis in the O-RA app, the *React-Native-tts* and *Expo-speech* packages were used to synthesize Thai-language voice instructions within the app, enhancing ease of use and convenience for older adult users.

### System Testing

The O-RA app was developed iteratively with input from clinical experts, including physiotherapists and rehabilitation physicians. Prior to the trial, a pilot test was conducted with a small group of users to identify and resolve critical usability issues. However, a formal usability evaluation using validated instruments (eg, System Usability Scale or Technology Acceptance Model) was not conducted prior to the trial, which is acknowledged as a limitation of this study. The app includes short exercise demonstration videos and step-by-step diagrams to guide correct exercise performance. A notification system during exercising is also included to remind users to perform their exercises. We plan to include more customizable reminder options, such as opt-in text messages at user-chosen intervals (eg, daily, 3 times per week), in future versions.

### Outcome Determination

The exercise program was considered complete when all 4 exercises were performed (on both sides for knee extension, leg curl, and hip abduction, and 2 times for semi-squat) at least 8 out of 10 times for each exercise. The correctness was measured according to the case record form and by calculating the score and percentage of the score achieved. The number of times and percentages of exercises were compared, and the scores and percentages of correctness of exercises were compared between the group using the O-RA app and the group using standard teaching methods. The VAS for pain during the exercise program was assessed as an adverse effect outcome. Satisfaction with and difficulty using the O-RA app were rated by the participants in the intervention group using a structured questionnaire with a VAS (0‐100), and obstacles to using the system were also collected through open-ended narrative questions.

### Statistical Analysis

Descriptive statistics, including frequencies and percentages for categorical variables or means (SDs) and medians (IQRs) for continuous variables, were used to describe demographic characteristics such as age, gender, and underlying diseases and other relevant variables of the study participants.

For continuous outcomes (compliance with the exercise program indicated by number of times and percentage of exercises, correctness of the exercise program indicated by exercise accuracy scores and percentage, pain VAS), inferential statistics were used. First, a Shapiro-Wilk test was applied to both outcomes to determine whether the distributions were normal. An independent *t* test was used if the parameter was normally distributed, whereas the Mann-Whitney *U* test was used if the parameter was not normally distributed. The Fisher exact test was used to compare categorical data, including the number of participants who exercised at least 80% of the time every day, the number of participants who correctly exercised, and the number of participants who experienced pain (as a safety monitoring measure), between the O-RA app group and the standard teaching group. For variables with non-normal distributions (as determined using the Shapiro-Wilk test), the median and IQR were reported; for normally distributed variables, the mean and SD were reported. The 2-tailed significance level was set at *P*<.05. The data were analyzed using an intention-to-treat method with a superiority design.

### Ethical Considerations

This study was conducted in accordance with the ethical standards of the responsible institutional and national committees on human experimentation and with the World Medical Association Declaration of Helsinki (1975, as revised in 2013). The study protocol was approved by the Institutional Ethics Committee of the Faculty of Medicine, Chiang Mai University, Chiang Mai, Thailand (study code: REH- 2566‐0329).

Written informed consent was obtained from all participants prior to randomization and data collection. Before providing consent, prospective participants received verbal and written information from a member of the research team describing the purpose of the study, exercise intervention procedures, expected duration of involvement, potential benefits and risks, data handling arrangements, and right to withdraw at any time without penalty. Caregivers responsible for maintaining participants' exercise logbooks were informed of the study procedures and voluntarily agreed to their role.

All participant data were handled in accordance with the data protection policies of the Faculty of Medicine, Chiang Mai University, and with the Thai Personal Data Protection Act B.E. 2562 (2019) [15]. Study records were pseudonymized using participant identification codes at the point of data entry; the key linking identifiers to codes were stored separately from the study dataset in a locked, access-restricted location accessible only to named members of the research team. Paper case record forms and exercise logbooks were stored in locked cabinets within the Department of Rehabilitation Medicine, Maharaj Nakorn Chiang Mai Hospital. Electronic data collected through the O-RA app were transmitted over encrypted connections and stored on Firestore and Firebase Storage (Google Cloud Platform), with access restricted to named investigators and developers via Firebase Authentication. Where identifiable information is present in figures, we collected informed consent from the individual.

Each participant received ฿300 (US $9.21) as compensation for travel expenses associated with the in-person study visits.

## Results

### Recruitment of the Participants

**Figure 3.**
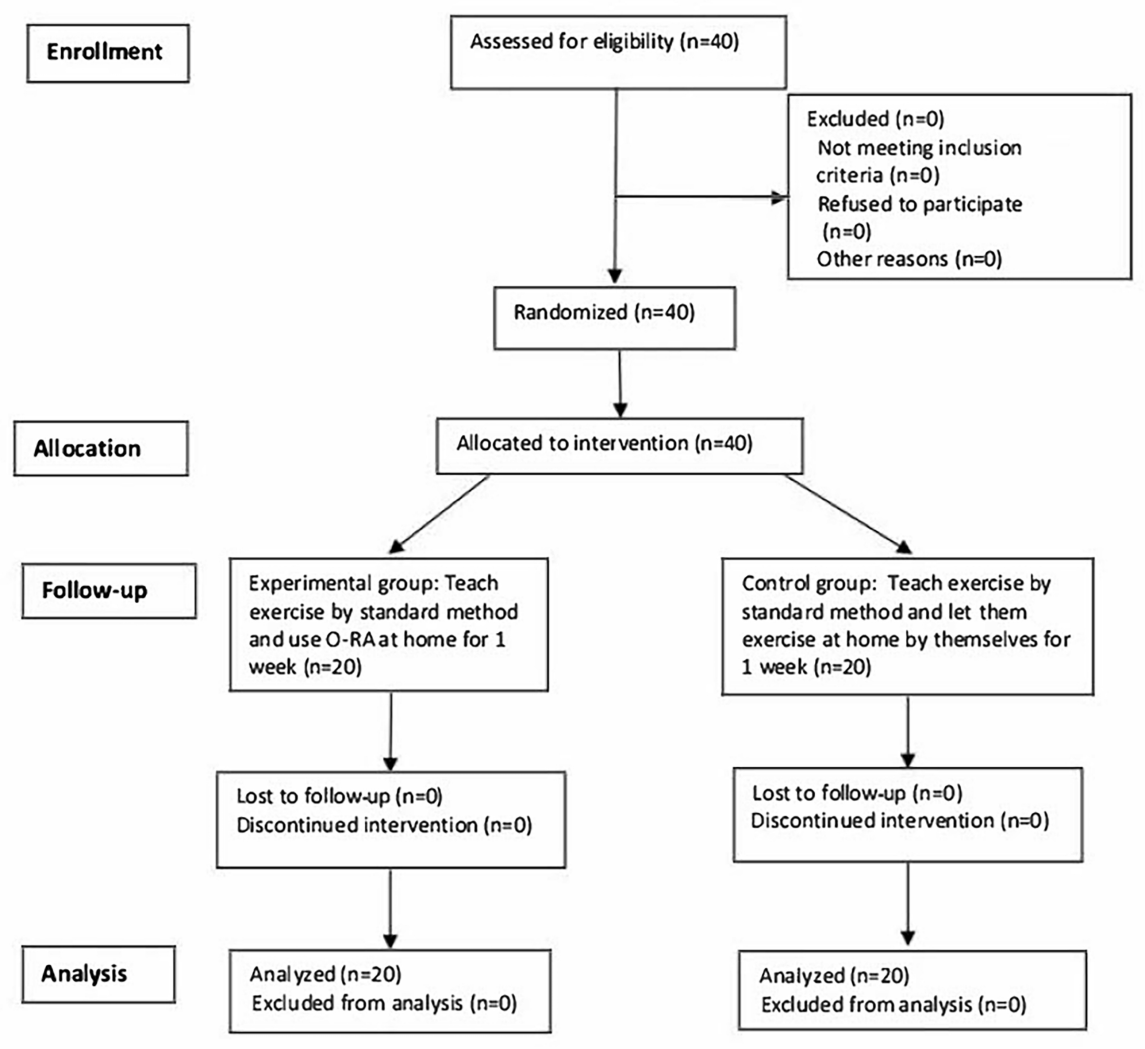
Study flow according to the CONSORT (Consolidated Standards of Reporting Trials) diagram. O-RA: Osteoarthritis-Rehabilitation Assistant.

During the 11-month data collection period, 40 participants were recruited. All participants completed the study protocol without loss to follow-up, and data from all participants were analyzed. Since there were no missing data, no imputation method was used. The CONSORT (Consolidated Standards of Reporting Trials) study flow diagram is presented in Figure 3.

### Baseline Characteristics of the Participants

Of the 40 recruited participants, the mean (SD) age was 67.2 (5.8) years. Most were female (33/40, 83%). The most common underlying disease was hypertension (20/40, 50%), followed by dyslipidemia (17/40, 43%). One-half of the participants had an education level higher than secondary school level (20/40, 50%), and 9 participants (9/40, 23%) were still working. When comparing the intervention and control groups, there were no statistically significant differences in age, sex, underlying disease, education level, and working status (Table 1).

**Table 1.** Baseline characteristics of the participants, with the significance level set at *P*<.05.

Characteristics	Overall (n=40)	Intervention group (n=20)	Control group (n=20)	Statistical test result (*df*)	*P* value
Age (years), mean (SD)	67.2 (5.8)	67.6 (6.0)	66.9 (5.6)	–0.0800 (38)^a^	.71
Sex (female), n (%)	33 (83)	17 (85)	16 (80)	—^b^	>.99
Underlying disease, n (%)					
Hypertension	20 (50)	9 (45)	11 (55)	—^b^	.75
Diabetes	8 (20)	3 (15)	5 (25)	—^b^	.70
Dyslipidemia	17 (43)	7 (35)	10 (50)	—^b^	.52
Others	13 (33)	6 (30)	7 (35)	—^b^	>.99
Highest education level, n (%)				—^b^	.48
No education	2 (5)	0 (0)	2 (10)		
Primary school	9 (22)	4 (20)	5 (25)		
Secondary school	7 (7)	3 (15)	4 (20)		
Higher degree	22 (55)	13 (65)	9 (45)		
Currently working, n (%)	9 (23)	5 (25)	4 (20)	—^b^	>.99

a Independent *t* test.

b 2-tailed Fisher exact test.

### Effects of the O-RA App on Compliance With the Exercise Program

Compliance with the exercise program was assessed using 3 outcome parameters, including the number of exercises per week, the percentage of observed number of exercises to the prescribed number of exercises, and the number of participants who completed at least 80% of the exercises every day. Notably, we used the percentage between the observed number of exercises and the prescribed number of exercises as a primary outcome since it was an outcome presented in the previous study we used as a reference for calculating the sample size [10].

Focusing on the primary outcome, participants in the intervention groups had significantly lower observed/prescribed number of exercise percentages than those in the control group for each type as well as all types of exercise (mean compliance: intervention, 75%, SD 22%; control, 94%, SD 8%; *t*_38_=3.5044; *P*=.001). We also found that participants in the intervention group had a significantly lower number of exercises per week than those in the control group for each type as well as all types of exercise (intervention: mean 1264, SD 373; control, mean 1575, SD 135; *t*_38_=3.5044; *P*=.001). In addition, the number of participants who completed all exercises every day, at least 80% of the time, was significantly lower in the intervention group than in the control group for hip abduction (intervention, 3/20, 15%; control, 10/20, 50%; *P*=.04) and leg curl (intervention, 3/20, 15%; control, 10/20, 50%; *P*=.04) exercise types but not significantly different for knee extension and semi-squat exercise types or for all types of exercise (Table 2).

**Table 2.** Comparison of the compliance outcomes between groups, with the significance level set at *P*<.05.

Outcomes	Overall (n=40)	Intervention group (n=20)	Control group (n=20)	Statistical test result (*df*)	*P* value
Compliance (number of exercises per week), mean (SD)					
Hip abduction (n=420)	360 (76)	323 (90)	397 (31)	3.5007 (38)^a^	.001
Leg curl (n=420)	358 (80)	318 (95)	397 (31)	3.5246 (38)^a^	.001
Knee extension (n=420)	356 (80)	322 (91)	390 (48)	2.9709 (38)^a^	.005
Semi-squat (n=420)	346 (93)	301 (109)	319 (40)	3.4567 (38)^a^	.001
All types of exercise (n=1640)	1420 (318)	1264 (373)	1575 (135)	3.5044 (38)^a^	.001
Compliance (%; observed number of exercises/prescribed number of exercises × 100), mean (SD)					
Hip abduction	86 (18)	77 (21)	95 (7)	3.5007 (38)^a^	.001
Leg curl	85 (19)	76 (23)	95 (7)	3.5426 (38)^a^	.001
Knee extension	85 (19)	77 (22)	93 (12)	2.9709 (38)^a^	.005
Semi-squat	82 (22)	72 (26)	93 (10)	3.4567 (38)^a^	.001
Overall compliance	85 (19)	75 (22)	94 (8)	3.5044 (38)^a^	.001
Compliance (number of participants who completed at least 80% of all exercises every day), n (%)					
Hip abduction	13 (33)	3 (15)	10 (50)	—^b^	.04
Leg curl	13 (33)	3 (15)	10 (50)	—^b^	.04
Knee extension	12 (30)	3 (15)	9 (45)	—^b^	.08
Semi-squat	12 (30)	3 (15)	9 (45)	—^b^	.08
All exercises	12 (30)	3 (15)	9 (45)	—^b^	.08

a Independent *t* test.

b 2-tailed Fisher exact test.

### Effects of the O-RA App on Correctness of Following the Exercise Program

Correctness of the exercise program was assessed using the correctness score and the number of participants who correctly exercised for each type as well as all types of exercise. We found no statistically significant differences in the correctness score and the number of participants who correctly exercised during each type of exercise as well as all types of exercise between the intervention and control groups (Table 3).

**Table 3.** Comparison of the correctness and pain outcomes between groups, with the significance level set at *P*<.05.

Outcomes	Overall (n=40)	Intervention group (n=20)	Control group (n=20)	Statistical test result (*df*)	*P* value
Correctness score (maximum=100), mean (SD)					
Hip abduction	80 (30)	83 (29)	78 (30)	3.5007 (38)^a^	.60
Leg curl	85 (26)	85 (29)	85 (24)	3.5246 (38)^a^	>.99
Knee extension	88 (27)	85 (33)	90 (21)	2.9709 (38)^a^	.59
Semi-squat	86 (25)	83 (29)	90 (21)	3.4567 (38)^a^	.36
Overall correctness	85 (21)	84 (26)	86 (15)	3.5044 (38)^a^	.78
Correctness (number of participants who correctly exercised), n (%)					
Hip abduction	26 (65)	14 (70)	12 (60)	—^b^	.74
Leg curl	29 (73)	15 (75)	14 (70)	—^b^	>.99
Knee extension	32 (80)	16 (80)	16 (80)	—^b^	>.99
Semi-squat	30 (75)	14 (70)	16 (80)	—^b^	.72
All exercises	19 (48)	11 (55)	8 (40)	—^b^	.53
Pain VAS^c^, median (IQR)	0 (0-0)	0 (0-0)	0 (0-0)	192.5^d^	.72
Participants who experienced pain, n (%)	5 (13)	3 (15)	2 (10)	—^b^	>.99

a Independent *t* test.

b 2-tailed Fisher exact test.

c VAS: visual analog scale.

d Mann-Whitney *U* test.

### Adverse Effects, Satisfaction, and Difficulty With Using the O-RA App

Adverse effects of exercise were assessed using the pain VAS during the week of performing the exercise program and the number of participants who experienced pain. We found no differences in median pain VAS between the intervention and control groups (intervention, 0; control, 0; *U*=192.5; *P*=.73). In addition, there was no difference in the numbers of participants who experienced pain between the intervention and control groups (3/20, 15% vs 2/20, 10%; *P*>.99; Table 3).

**Table 4.** Difficulty and satisfaction with using the Osteoarthritis-Rehabilitation Assistant (O-RA) app (n=20).

Parameters	Results, mean (SD)		Results, median (IQR)		Results, range	
Satisfaction	47 (32)		43 (22-77)		0-100	
Difficulty	59 (32)		60 (38-84)		0-100	

According to the satisfaction level and feasibility of the O-RA app for participants in the intervention group, the median satisfaction with the O-RA app was 43 (IQR 22-77), and the median difficulty using the O-RA app was 60 (IQR 38-84; Table 4). Notably, participants in the intervention group who exercised every day, at least 80% of the time (n=3), had a lower difficulty score than those who did not (n=17; median difficulty score: 30 vs 65). No statistical test was conducted due to a very small sample size (n=3) in one group.

Focusing on narrative comments on the use of the O-RA app, two participants reported that the app was not stable (could not be opened); 3 participants reported that it wrongly detected their body motions, which caused too many repetitions of the exercise; and 2 participants complained that the app requires a continuous WiFi internet connection during the entire exercise program. However, their WiFi internet connection was not stable, which made the exercise program incomplete. Overall, 8 participants (8/20, 40%) indicated that it was too difficult for them to use the O-RA app as a routine exercise assistant.

## Discussion

### Principal Findings

This study aimed to demonstrate the effect of the O-RA app on increasing compliance and correctness of the exercise program for knee OA. Since this is the first study using the O-RA app with humans, we conducted this assessor-blinded, randomized controlled clinical trial with older adults who had not been diagnosed with and had no symptoms of knee OA for safety purposes. Therefore, this study should be considered as a phase 1 or early exploratory study. We hypothesized that the O-RA app can increase the compliance with and correctness of the exercise program. Contrary to our hypothesis, results from this study demonstrated that the O-RA app was not a facilitator but a barrier to the exercise program. In addition, the correctness of the exercise program was not different between the groups that used and did not use the O-RA app. These unexpected results indicate that this version of the O-RA app may not be appropriate for improving exercise adherence and an improved version should be developed. Beyond the instability and the requirement for continuous WiFi connectivity, participants also reported difficulty navigating the interface, lack of familiarity with smartphone technology, and a preference for in-person physiotherapy guidance as additional reasons for reduced app usage. A formal usability assessment using standardized instruments (eg, System Usability Scale) would be valuable for systematically characterizing these barriers in future studies.

The explanation for why participants who used the O-RA app had lower compliance with the exercise program than participants who did not use it may be associated with the difficulty using the app. Although the developers of the O-RA app tested its usability in their proof-of-concept experiment, it was never tested in a clinical setting before this study. In a real-life clinical setting, users of the app are varied in many aspects, including knowledge, skills, and experiences regarding mobile app use, which may cause difficulty when using the mobile app to facilitate exercise program compliance. This problem may even worsen exercise adherence by making the participant frustrated. Notably, optimal usability study design involves recruiting end users with variable skills and knowledge levels to obtain broad-based feedback from both tech-savvy and tech-challenged users. This explanation is confirmed by the results of this study, which demonstrated that participants in the intervention group who completely exercised at least 80% every day had a lower difficulty score than those who did not complete the exercises. The main problems that made the use of the O-RA app difficult were (1) stability of app access, (2) invalidity of movement detection, (3) requirement for a continuous WiFi internet connection, and (4) overall difficulty for Thai older adults. Therefore, the developers of this app should consider these pain points and improve the app accordingly. For example, built-in data storage in the app instead of using cloud storage should be considered to increase the stability of app access and make the app usable without a continuous WiFi internet connection. In addition, a user-friendly UX/UI system should be specifically developed to facilitate use by Thai older adults, such as large font size and easy-to-understand calibration processes and performance steps. Additionally, options should be built into the app to accommodate users with variable levels of health literacy and technical capability, such as a simplified navigation mode and multilingual support.

Focusing on the effect of each exercise type, a trend of increased efficacy of the intervention for more difficult exercise types was observed. For example, the intervention group had a significantly lower percentage of participants who exercised at least 80% of the time every day for hip abduction and leg curl but not for knee extension and semi-squat exercises, resulting in a nonsignificant difference in the overall exercise program. This discrepancy may indicate that the O-RA app should be more effective at improving compliance with the more complicated exercises. Most of the exercises used in this study are relatively easy; therefore, the participants can perform them without problems. Adding the use of the O-RA app to these easy-to-perform exercises may make the exercise activities more complicated and redundant. Therefore, it may not increase but even decrease compliance with the exercise program, causing lower compliance in the intervention group than in the control group.

According to the correctness outcomes, since the exercise program was relatively easy, no effect of the O-RA app was observed since the correctness reached the ceiling in the control group. It may be both interesting and more valuable to add only the O-RA app to complicated exercises, such as semi-squat exercises, back extension exercises, or back flexion exercises, to make the potential positive effect of the O-RA app more evident. In addition, no significant increase in pain was observed in the intervention group when compared with the pain in the control group. This result can confirm the safety of O-RA app use by older adults who have no diagnosis and no symptoms of knee OA. However, since the safety outcome was not the primary outcome, the sample size calculated from the efficacy outcome is likely inadequate, which may cause an underpowered safety result. Therefore, adverse effects of using the O-RA app should be monitored in future studies.

When compared with results from previous studies, there were some differences since most of them demonstrated significant effects of mobile apps for facilitating an exercise program. These inconsistencies may be due to the differences in study methodology as well as the mobile app used between this study and the previous ones. For example, the study by Alasfour and Almarwani [10], which demonstrated a significant improvement in adherence to the exercise program between the mobile app group and the handout group, used a mobile app that did not include motion analysis. The study by Thiengwittayaporn et al [12], which demonstrated a significant improvement in the correctness of the exercises between the mobile app group and the handout group, also used a mobile app that did not include motion analysis. We hypothesized that, although motion analysis is a powerful tool for creating a personalized exercise program, its complicated function may make the users, especially those who are not familiar with this technology, frustrated, causing unfavorable compliance and correctness outcomes. On the other hand, the study by Biebl et al [11] demonstrated a noninferior effect on the correctness of the exercises in the motion analysis–based mobile app group and physiotherapist-supervised group. This inconsistency may be due to the difference in the mobile app used between the studies. In the study by Biebl et al [11], the motion analysis mobile app was easier to use than our mobile app since it required no calibration at the beginning of each exercise session. In addition, the exercise program in the study by Biebl et al [11] was more complicated than the program in our study. Therefore, motion analysis feedback may add some value. These issues address the importance of the feasibility of mobile apps and the selection of an exercise program where motion analysis should be added.

### Strengths and Limitations

We would like to appraise our study and discuss its strengths and limitations using the GRADE (Grading of Recommendations, Assessment, Development, and Evaluations) paradigm [16]. According to this paradigm, 5 issues relevant to this study that affect the certainty or quality of evidence should be considered, including study design, risk of bias, imprecision, inconsistency, and indirectness of the evidence. Its strength is related to the study design, which is a phase 1 randomized controlled trial providing preliminary evidence with a relatively high level of certainty and quality. As a preliminary finding, the effect size of the primary outcome has a small SD, although the precision of these estimates should be confirmed in a larger phase 2 trial. However, the results are internally consistent since the effects of the intervention on all outcomes were in the same direction (ie, favoring the control), which indicates a problem regarding the use of the O-RA app. Notably, as a phase 1 trial, these findings are exploratory and should be interpreted with caution.

According to the limitations of this study, first, potential biases in this study should be discussed since they may introduce systematic error that makes the results deviate from the truth. The first issue is selection bias (ie, unequal baseline characteristics between the comparison groups). Since this study used a valid randomization method (simple randomization) and allocation concealment (a sealed envelope) and the statistical tests demonstrated that baseline characteristics were not different between the groups, there may not be problematic selection bias in this study. The second issue is performance bias, both for participants and investigators. In this study, the investigators (the physiotherapist and rehabilitation physician) but not the participants were blinded to the study group, which may cause a risk of performance bias from the participant side. For example, participants who knew that they were in the control group tried to complete the exercise program as much as possible since they wanted to be a responsible person from the perspectives of health care providers. This behavior, known as the Hawthorne effect, may cause a higher increase in exercise compliance in the control group, resulting in performance bias that reduces the effect size. For the detection bias, since this study mostly used the objective outcome (compliance was evaluated by the family member, and correctness was assessed by the investigator), there may be a low risk of detection bias. Since this study has no missing participants and all outcomes were reported according to the protocol, it may have a low risk of attrition and reporting bias. Therefore, this study may have a risk of performance bias from a lack of participant blinding. However, it is difficult to blind the participants of an exercise intervention [17]. One potential approach to mitigate this limitation is to have the control group perform sham exercises, which is a method commonly used in exercise intervention trials to reduce performance bias. Next, according to the indirectness issue, the study population of this study was older adults without knee OA symptoms or diagnosis. Therefore, the results cannot be generalized to older adults who already have knee OA symptoms or diagnosis. Performing exercises by a population with the disease may be more challenging than by those without disease; therefore, further studies should be conducted in the target population. Additionally, this study did not use a standardized app usability evaluation tool (eg, the System Usability Scale or Technology Acceptance Model), which limits our ability to systematically characterize usability barriers. Future iterations of the study should incorporate such validated instruments.

### Clinical and Research Implications

For clinical implications, we suggest not using this version of the O-RA app for increasing compliance with a lower extremity exercise program in older adults with knee OA symptoms or diagnosis. Since there is no direct evidence, we also suggest not using this version of the O-RA app for increasing compliance with the exercise program in older adults who already have knee OA symptoms or a diagnosis.

For research implications, an improved version of the O-RA app should be developed, one that is more stable, more user-friendly, and easier to use. After that, a study aiming to evaluate the usability and reliability of the new version of the O-RA app should be conducted before any further clinical trials. In addition, further studies aiming to adjust the O-RA app to assist users performing more complicated exercise programs should be considered. Further studies should also be conducted in another target population, such as those who already have knee OA symptoms or diagnosis, before using the O-RA app, preferably an updated one, with that specific population.

### Conclusions

Results from this randomized controlled parallel trial demonstrate that O-RA, a motion analysis–based mobile app, was not a facilitator but a barrier for older adults without knee OA symptoms or diagnosis to complete the lower extremity exercise program. This result may be due to the instability of and difficulty using the app. An updated version, aiming to increase the stability and be more user-friendly, should be developed. More comprehensive data, including qualitative user feedback and standardized usability metrics (eg, System Usability Scale), should be collected to effectively guide the design of future versions.

## Supplementary material

10.2196/80971 Checklist 1 CONSORT-EHEALTH checklist.

## Abbreviations

CONSORT: Consolidated Standards of Reporting Trials
CONSORT-EHEALTH: Consolidated Standards of Reporting Trials of Electronic and Mobile Health Applications and Online Telehealth
CPU: central processing unit
CSS: cascading style sheets
GPU: graphics processing unit
GRADE: Grading of Recommendations, Assessment, Development, and Evaluations
mHealth: mobile health
O-RA: Osteoarthritis-Rehabilitation Assistant
OA: osteoarthritis
UI: user interface
UX: user experience
VAS: visual analog scale
